# Intramural metastasis of T1 rectal cancer: report of a case report

**DOI:** 10.1186/s12957-015-0749-5

**Published:** 2015-12-16

**Authors:** Kosuke Toda, Kenji Kawada, Suguru Hasegawa, Masahiro Yamada, Junichiro Kawamura, Yoshiharu Sakai

**Affiliations:** Department of Surgery, Graduate School of Medicine, Kyoto University, 54 Shogoin- Kawara-cho, Sakyo-ku, 606-8507 Kyoto Japan; Department of Surgery, Shiga Medical Center for Adults, Moriyama, Japan; Department of Surgery, Faculty of Medicine, Kinki University, Osaka Sayama, Japan

**Keywords:** Rectal cancer, Intramural metastasis, Submucosal tumor

## Abstract

**Background:**

Intramural metastasis (IM) is extremely rare in colorectal cancer, although it often occurred in esophageal cancer.

**Case Presentation:**

We report a rare case of T1 rectal cancer with IM which was successfully resected by laparoscopic surgery. A 62-year-old man was admitted to our institution for the treatment of rectal cancer detected by medical examination. Colonoscopy revealed two tumors in the rectum: a type II rectal cancer of 2 cm in diameter located 5 cm proximal to the anal verge and a submucosal tumor of 1 cm in diameter located approximately 1.5 cm proximal to the rectal cancer. Abdominal computed tomography (CT), magnetic resonance imaging (MRI), and transrectal ultrasonography indicated the rectal cancer invaded into the submucosal layer with no metastasis to regional lymph nodes or distant organs. The patient underwent laparoscopic intersphincteric resection.Histopathological analysis revealed that the rectal cancer was moderately differentiated adenocarcinoma (stage I; pT1N0M0 according to the 7th edition of UICC) with severe lymphovascular invasion (ly1, v3) and that the submucosal tumor was composed of moderately differentiated adenocarcinoma proliferating within the muscularis propria. A number of features of the submucosal tumor indicated that this was an IM of the rectal cancer: clearly distinct location from the rectal cancer, growth predominantly within the muscularis propria, similar structural and cellular heterogeneity, and the presence of tumor emboli within vascular vessels. The patient was postoperatively followed for more than 4 years without any sign of recurrence.

**Conclusions:**

To the best of our knowledge, this is the first report of the T1 rectal cancer with IM.

## Background

There have been few reports of intramural metastasis (IM) of rectal cancer, although IM has been frequently reported and considered to be one of the most important prognostic factors in esophageal cancer [1–3]. Consequently, it is unclear about the clinical significance and the treatment strategy of IM in rectal cancer. We believe this is the first report of the T1 rectal cancer with IM.

## Case presentation

**Fig. 1:**
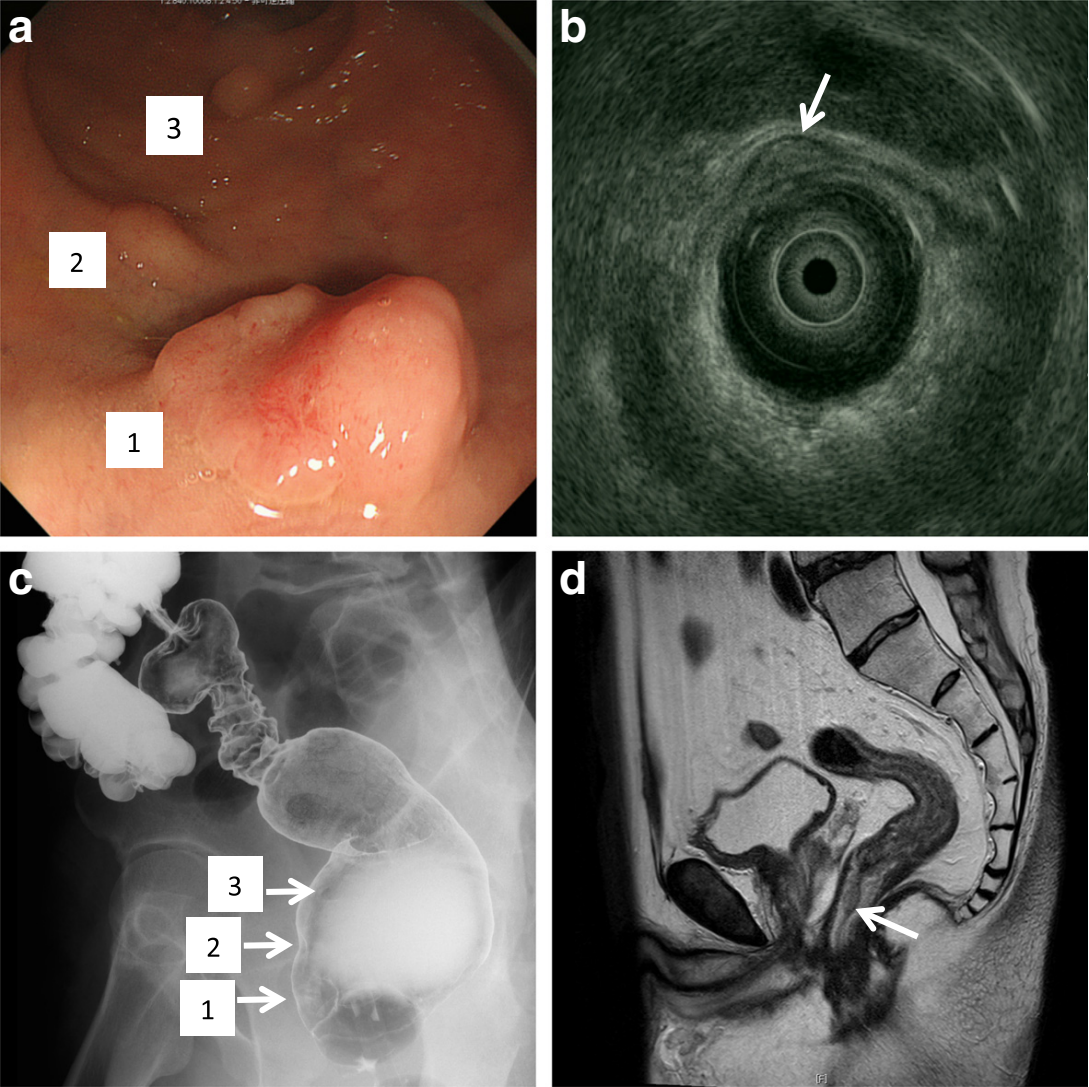
**a** Colonoscopy revealed a primary rectal cancer (*1*), a submucosal tumor (*2*), and an adenomatous polyp (*3*). **b** Transrectal ultrasonography showed that the invasion depth of rectal cancer was the deep layer of the submucosa but not the muscularis propria. **c** Barium enema examination showed that a rectal cancer with an irregular surface (*1*), a submucosal tumor with a smooth surface (*2*), and an adenomatous polyp (*3*) are located at the anterior wall of the rectum. **d** MRI showed that the rectal cancer was located at the anterior wall of the rectum without invasion into the prostate

A 62-year-old man was admitted to our hospital for the treatment of rectal tumor incidentally found by rectal examination during a routine medical checkup. The patient had no previous history of malignancy. Colonoscopy revealed a type II rectal tumor of 2 cm in diameter located 5 cm proximal to the anal verge and a submucosal tumor of 1 cm in diameter approximately 1.5 cm proximal to the rectal cancer. In addition, a small adenomatous polyp was observed near the rectal cancer (Fig. 1a). The biopsy of the rectal tumor was suggestive of moderately differentiated adenocarcinoma. The biopsy of the submucosal tumor was not performed because it was thought to be included within the region planned for surgical resection against the rectal cancer. Transrectal ultrasonography suggested the invasion depth of rectal cancer was the deep layer of the submucosa but not the muscularis propria (Fig. 1b). Abdominal CT showed there was no evidence of metastasis to regional lymph nodes or distant organs (data not shown). Barium enema examination suggested a small tumor with an irregular surface and a smaller submucosal tumor with a smooth surface located at the anterior wall of the rectum (Fig. 1c). MRI showed that the rectal cancer was located at the anterior wall of the rectum without invasion into the prostate (Fig. 1d), and diffusion-weighted imaging (DWI) that showed a high signal intensity was accumulated into the rectal cancer (data not shown). Results of laboratory blood tests, including tumor markers, were within normal ranges. Collectively, we preoperatively diagnosed cStage I rectal cancer and performed laparoscopic intersphincteric resection (ISR).

**Fig. 2:**
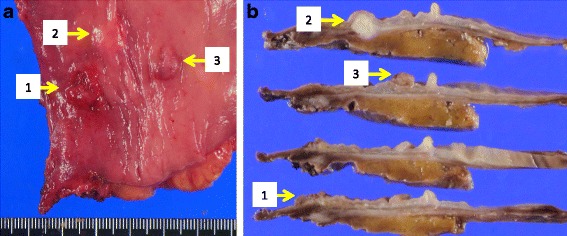
Resected rectal tumors. **a** Macroscopic findings; a 16 × 15-mm type II rectal cancer (*1*), a 10 × 10-mm submucosal tumor (*2*), and a 10 × 7-mm adenomatous polyp (*3*). **b** Gross appearance of cross-section showed a primary rectal cancer (*1*), a submucosal tumor (*2*), and an adenomatous polyp (*3*). Submucosal tumor (*2*) was not connected to the primary rectal cancer (*1*) and was located within muscularis propria

**Fig. 3:**
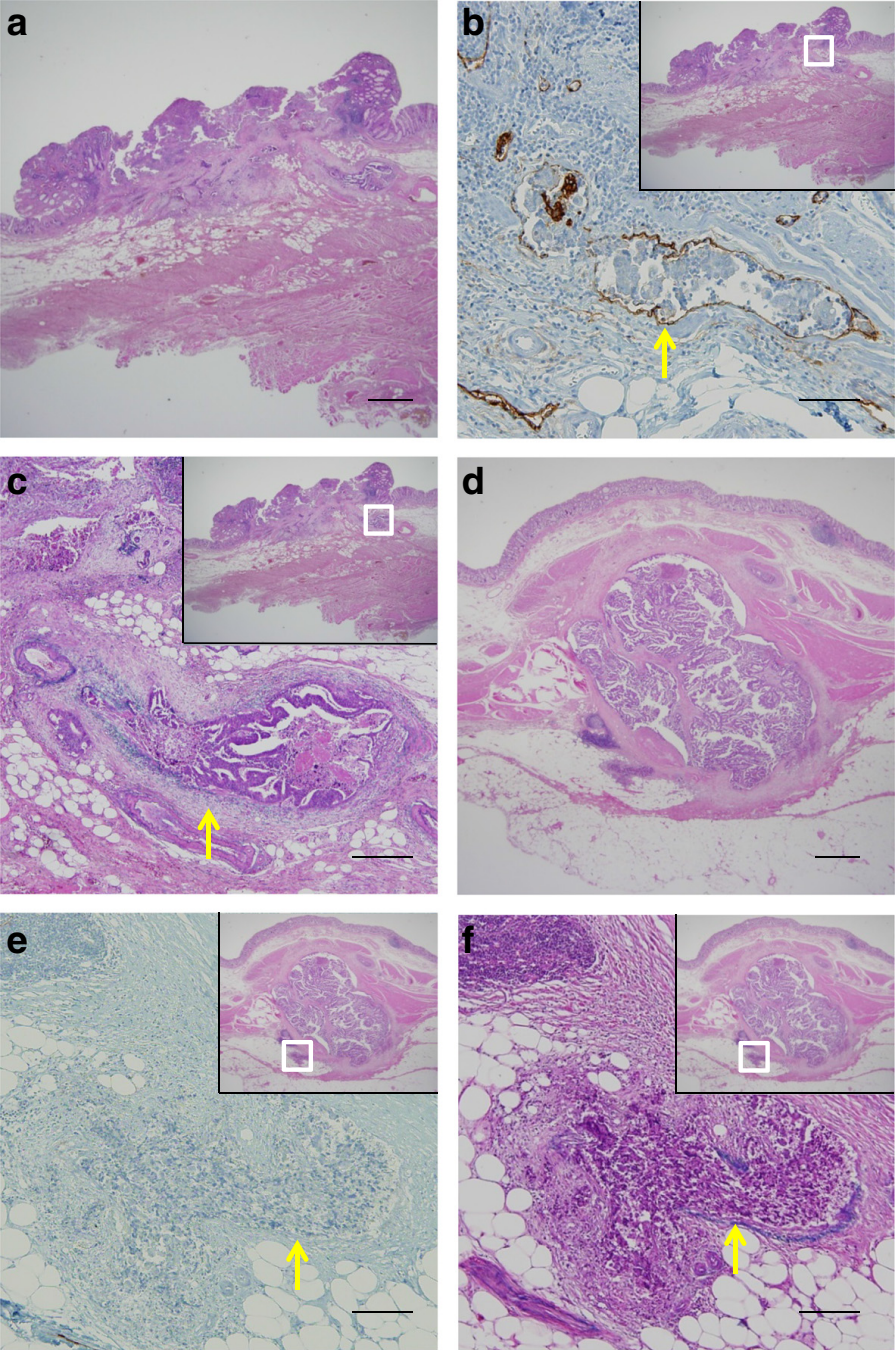
Histological findings (**a**, **d** H&E, ×12.5, scale bar, 1000 μm; **b**, **e** D2-40 staining, ×100, scale bar, 100 μm; **c**, **f** Victoria blue staining, ×100, scale bar, 100 μm) **a**–**c** The primary rectal cancer was moderately differentiated adenocarcinoma with severe lymphovascular invasion: pT1 (submucosal invasion depth 3500 μm), ly1, v3. **d**–**f** The submucosal tumor was composed of moderately differentiated adenocarcinoma proliferating within the muscularis propria, and the tumor emboli within vascular, not lymphatic, vessels, were observed around the submucosal tumor

Macroscopic analysis of resected specimens revealed a 16 × 15-mm type II rectal cancer with an irregular surface, a 10 × 10-mm submucosal tumor located 15 mm proximal to the primary rectal cancer, and a 10 × 7-mm adenomatous polyp located 20 mm proximal to the primary rectal cancer (Fig. 2a, b). Histopathological analysis revealed that the primary rectal cancer was moderately differentiated adenocarcinoma with severe lymphovascular invasion: pT1 (submucosal invasion depth 3500 μm), pN0, pM0, ly1, v3, and pStage I. Immunohistochemical analysis using the stainings of D2-40 and Victoria blue was performed to evaluate lymphatic and vascular invasion (Fig. 3a–c). The submucosal tumor was composed of moderately differentiated adenocarcinoma proliferating within the muscularis propria. Moreover, numerous tumor emboli within vascular, but not lymphatic, vessels were observed in regions surrounding the submucosal tumor (Fig. 3d–f). We diagnosed the submucosal tumor to be an IM of the primary rectal cancer based on the following observations: (1) distinct location from the rectal cancer, (2) the gross appearance of the submucosal tumor without intraepithelial cancer extension, (3) the same histological type as the primary rectal cancer, and (4) tumor emboli within vascular vessels observed in regions surrounding the submucosal tumor as well as the primary rectal cancer. Three weeks after the operation, the patient was discharged without any event. The stage of the rectal cancer was stage I, but the patient elected to undergo adjuvant therapy because IM is one of the poorer prognostic factors in esophageal and gastric cancers [1–4]. Adjuvant chemotherapy (UFT + LV) was administered for 6 months postoperatively. The patient has been followed for more than 4 years without any signs of recurrence.

### Discussion

IM is often observed in esophageal cancer but quite rarely in rectal cancer. IM of the esophageal cancer was first reported by Watson in 1933 [5], and the frequency of IM has been reported to be about 10–15 % in advanced esophageal cancers [1–3]. The prognosis in esophageal cancer with IM was poor because of higher frequencies of lymph node and distant metastases [1–3]. In rectal cancer, lymphovascular invasion beyond the primary lesion is termed to be microscopic distal intramural spread (DIS). According to previous studies, DIS occurs in 10–40 % of rectal cancer, is significantly associated with lymph node and distant metastases, and is a risk factor for local recurrence and poor prognosis [6–8]. When DIS occurs, it is usually within 2.0 cm of the tumor [6–8]. As a result, a 2-cm distal margin has become acceptable for resection of rectal cancer. IM in esophageal cancer has been interpreted to be caused by lymphovascular invasion into the submucosal layer. In the present case, histological findings demonstrated invasion of the rectal cancer into the submucosal layer (T1) with severe lymphovascular invasion, a submucosal tumor consisting of cancer cells within the muscularis propria, and the tumor emboli within vascular vessels surrounding the submucosal tumor. Therefore, the submucosal tumor was assumed to represent IM via vascular invasion of tumor cells. To the best of our knowledge, this is the first report of T1 rectal cancer with IM.

The management of rectal cancer has become increasingly complex. Presently, three major curative surgical interventions are available: local excision, sphincter-preserving surgery, and abdominoperineal resection. Indications for local excision that preserves anal sphincter anatomy and function include small T1 lesions. However, the use of local excision is limited by an inability to assess regional lymph nodes and uncertainty of oncologic outcome. According to guidelines issued by the Japanese Society for Cancer of the Colon and Rectum (JSCCR) in 2010, the criteria for identifying curable T1 colorectal cancer after endoscopic resection were well/moderately differentiated of papillary histologic grade, no vascular invasion, submucosal invasion depth less than 1000 μm, and tumor budding grade 1 (low grade) [9]. The local recurrence rate in patients with T1 rectal cancer following resection is in the range of 4–14 % at 5 years [10–12]. In the present case, sphincter-preserving surgery (i.e., laparoscopic ISR) was considered to be adequate considering the distance of submucosal invasion depth and severe lymphovascular invasion. The prognosis of esophageal cancer with IM is exceedingly poor with a survival rate of 9 % at 5 years and a median survival time of 0.7 years [2], which made IM one of the poor prognostic factors. Although there is a lack of consensus regarding treatment strategies, IM in rectal cancer may be a poor prognostic factor as in esophageal cancer. In the present case, postoperative adjuvant chemotherapy was done due to the patient’s desire. Regarding the strategy of rectal cancer with IM, further studies are required to facilitate the development of treatment strategies for rectal cancer with IM.

## Conclusions

We report a case of rectal cancer with IM. IM of rectal cancer is extremely rare; however, careful observation of the residual rectum around the primary tumor should be conducted, even in early rectal cancer. Preoperative diagnosis of IM may help to decide therapeutic strategy.

## Consent

Written informed consent was obtained from the patient for publication of this case report and any accompanying images. A copy of the written consent is available for review by the Editor-in-Chief of the journal.
